# Vortioxetine for the Treatment of Depression in Patients with Parkinson’s Disease: A 16-week Pilot, Prospective, Open-label Safety Study

**DOI:** 10.2174/011570159X361647250416072053

**Published:** 2025-06-02

**Authors:** Fabrizio Stocchi, Daniele Bravi, Fabiana Giada Radicati, Chiara Coletti, Laura Vacca

**Affiliations:** 1 Department of Neurology, University San Raffaele, Rome, Italy;; 2 Department of Neurology, Institute for Research and Medical Care IRCCS San Raffaele, Rome, Italy

**Keywords:** Vortioxetine, Parkinson’s disease, major depression, open-label, non motor symptoms, multimodal antidepressant

## Abstract

**Background:**

Parkinson’s disease (PD) is often associated with depression, which poses an additional burden for patients and their families. However, evidence regarding the optimal treatment for depression in PD remains limited, with insufficient data supporting the efficacy of most antidepressant drugs.

**Methods:**

The primary objective of this pilot, prospective, open-label, single-arm study was to analyze the safety and tolerability of vortioxetine drops on depressive symptoms in PD patients over 16 weeks of treatment. The secondary objective was to study vortioxetine's effectiveness on depression.

**Results:**

Sixteen out of 20 PD patients who completed the study demonstrated that the treatment was safe and well tolerated; no change in PD symptom severity, abnormality of clinical parameters, body weight, or ECG emerged. The most common side effect was nausea. Depressive symptoms rated by the Beck Depression Inventory and the Hamilton Depression Rating Scale score (HAM-D-17) showed a significant improvement at the end of the study period without a worsening of motor functions, as measured by UPDRS part III. The majority of patients also reported an improvement in depressive symptoms measured by the Patient Global Impression of Improvement scale.

**Conclusion:**

Vortioxetine is a safe and well-tolerated therapeutic approach for depression in Parkinson’s disease. As a secondary objective, an improvement in depressive symptoms was observed. However, the study’s open-label design and small sample size limit the generalizability of the findings.

**Clinical Trial Registration Number:**

NCT04301492.

## 1 INTRODUCTION

Depression is a frequent non-motor symptom (NMS) in both early and advanced stages of Parkinson’s Disease (PD) [1]. A systematic review found the weighted prevalence of major depressive disorder in patients with Parkinson’s disease to be 17%, minor depression 22%, and dysthymia 13% [2]. The occurrence of depression appears to be associated with an increased risk of late-stage complications such as dementia, falls, and caregiver distress [3]. Prevalence rates for depression in PD range between 10% and 90% [4, 5].

Depression can also precede the onset of motor symptoms and may play an important role in the clinical course of the disease. Its presence has been associated with greater disability, more rapid cognitive decline, increased mortality, and a heavier burden on families and caregivers [6-9].

Despite its profound impact on quality of life and cognitive functioning, depression in PD is often under-recognized and poorly treated [10]. Only 20% to 25% of PD patients with depression receive appropriate nursing and antidepressant therapies [11].

The mechanisms underlying depression in PD remain incompletely understood, but autopsy and neuroimaging studies have demonstrated alterations in the limbic system [12, 13], as well as in the noradrenergic and serotonergic nuclei of the brainstem [14]. This dual pathophysiology may explain the partial ineffectiveness of standard antidepressants, which often do not target dopaminergic dysfunction.

Vortioxetine is a multimodal antidepressant that acts as a 5-HT3, 5-HT7, and 5-HT1D receptor antagonist, a 5-HT1B partial agonist, a 5-HT1A receptor agonist, and a serotonin transporter (SERT) inhibitor. It modulates serotonergic receptor activity, inhibits serotonin reuptake, and influences other neurotransmitters such as norepinephrine, dopamine, acetylcholine, and histamine [15].

Currently, tricyclic antidepressants and selective serotonin reuptake inhibitors (SSRIs) are the most commonly used pharmacological treatments for depression in PD. However, results from clinical studies remain contradictory, and both drug classes have been reported to induce or exacerbate motor disability and other PD symptoms. Moreover, clinical efficacy has generally been modest [16].

Recent evidence suggests that the use of SSRI, but not other antidepressant classes, is associated with increased apathy in PD, further limiting their use, especially in patients with apathetic features and requiring alternative treatments [17].

Vortioxetine may represent an innovative pharmacological option for the treatment of depression in PD due to its ability to enhance dopaminergic neurotransmission in brain regions implicated in mood regulation. It also has a favorable safety profile in patients with major depression [18], along with a minimal risk of pharmacokinetic and pharmacodynamic drug interactions [19].

Vortioxetine is available in tablet form and as an oral solution (20 mg/mL). The oral drop formulation is particularly useful for dose titration and may be especially advantageous for PD patients with swallowing difficulties or who are on multiple medications.

The aim of the present prospective open-label single-arm study (VorDe-PD Study) was to evaluate the tolerability and safety of vortioxetine oral drops in PD patients with depressive symptoms. Secondary objectives included assessing the effectiveness of vortioxetine in reducing depressive symptoms.

## 2 MATERIALS AND METHODS

This was a pilot, single-center, open-label, intention-to-treat (ITT) study conducted at the IRCCS San Raffaele Pisana in Rome, Italy (NCT04301492), in accordance with Good Clinical Practice (GCP) guidelines, data protection laws, and the principles of the Declaration of Helsinki. The study was approved by the IRCCS San Raffaele Institutional Review Board in December 2019. All participants provided written informed consent prior to inclusion.

The study population included patients with idiopathic Parkinson’s disease and clinically significant depressive symptoms. Eligible patients were aged between 30 and 80 years, classified within Hoehn & Yahr stages 1 to 3, and had a Hamilton Depression Rating Scale (HAM-D-17) score ≥14 and a Beck Depression Inventory (BDI) score ≥13. All patients were required to be on stable doses of antiparkinsonian medications for at least 4 weeks prior to enrollment.

Exclusion criteria included atypical parkinsonism, cognitive impairment, high suicide risk (HAM-D-17 Item 3 score ≥3), presence of significant psychiatric, metabolic, or systemic diseases, clinically significant laboratory abnormalities, risk of pregnancy, history of epileptic seizures, Dopa Dysregulation Syndrome (DDS), use of irreversible monoamine oxidase inhibitors (MAOIs), and prior use of vortioxetine or other antidepressants within 30 days of study entry.

Treatment began with 3 drops/day (3 mg) administered after dinner, with weekly up-titration by 1 drop/day until reaching 10 drops/day (10 mg). Based on clinical evaluation, the dose could be increased to a maximum of 20 drops/day (20 mg).

The study included seven scheduled visits: V1 (baseline), V2 (week 1), V3 (week 2), V4 (week 4), V5 (week 8), V6 (week 12, telephone visit), and V7 (week 16, end of observational period). At baseline (V1), patients underwent assessment with the Hoehn & Yahr scale, Unified Parkinson’s Disease Rating Scale (UPDRS) part III, HAM-D-17, BDI, Patient Global Impression of Severity (PGI-S), and Patient Global Impression of Change (PGI-C). Safety was assessed through vital signs, laboratory tests, ECG, physical and neurological examinations, and treatment-emergent adverse events (TEAEs). Tolerability was assessed by comparing UPDRS scores at baseline and at the end of the study.

The primary endpoint was the safety and tolerability of vortioxetine after 16 weeks. Secondary endpoints included changes in depression severity as measured by HAM-D-17, BDI, PGI-S, and PGI-C scores.

Descriptive statistics were used for baseline demographics. The Kolmogorov-Smirnov test assessed variable normality. Paired Student’s t-tests were used to compare baseline and endpoint values. Results were reported as means ± standard deviation, and significance was set at *p*<0.05. Statistical analyses were conducted using SPSS version 28.0.

## 3 RESULTS

**Fig. (1):**
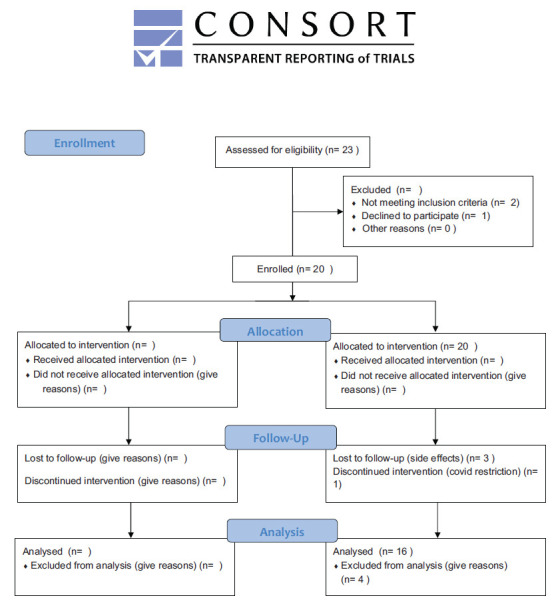
CONSORT 2010 flow diagram.

Twenty patients were enrolled, with 16 completing the study (Fig. **1**). Baseline demographics are shown in Table **1**.

Vortioxetine was generally safe and well tolerated. No clinically significant abnormalities in laboratory parameters, body weight, heart rate, or blood pressure were observed. ECG evaluations showed no meaningful changes, including in the QTcF interval.

A total of 5 adverse events (25%) were reported. Three patients experienced nausea, one of whom also had dizziness and confusion; all three discontinued treatment. One patient withdrew due to COVID-19-related restrictions, and one reported a decrease in libido, which did not require treatment discontinuation.

Among those completing the study, 13 patients reached 10 drops/day (10 mg), 2 required 15 drops/day (15 mg), and 1 required 20 drops/day (20 mg).

Statistically significant improvements in depressive symptoms were observed in both HAM-D-17 and BDI scores at the end of the study compared to baseline. Motor function, as assessed by the UPDRS-III, remained stable, indicating no worsening of Parkinsonian symptoms. Results are summarized in Tables **2**, **3** and Fig. (**2**).

**Fig. (3):**
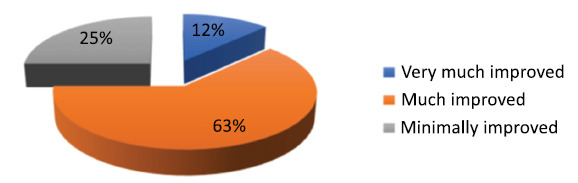
Patient global impression of change after 16 weeks of treatment.

Patient-reported outcomes *via* PGI-C also indicated subjective improvement in most cases (Fig. **3**).

## 4 DISCUSSION

Results from the present study demonstrated good safety tolerability of Vortioxetine in patients with PD. Most importantly, PD symptoms did not worsen, as shown by the UPDRS III evaluation, which remained unchanged. In line with the literature [18], gastrointestinal symptoms (nausea and/or vomiting) were the most frequent adverse event. Cardiovascular safety was also confirmed in terms of there being no effect on QT interval [18, 20].

The use of a slow titration with the drops formulation was associated with good tolerability and a lower incidence of side effects. Three patients dropped out of the study because of nausea, and one of these participants also experienced dizziness. Nausea is a common problem in PD patients because of slow gastric emptying and the use of dopaminergic drugs. Vortioxetine most probably exacerbated this symptom, but it only happened in 3 patients out of 20. Dizziness could be due to hypotension, which is another common problem in PD. The open-label nature of the study limits conclusions regarding causality.

The present open-label study confirms the results of a prospective, published study on vortioxetine in the treatment of depressive symptoms and other NMSs in PD, in which PD patients with major depression improved in depressive symptoms and apathy 3 months after starting with vortioxetine. Additionally, vortioxetine was safe and well-tolerated [21]. However, due to the small sample size and lack of blinding, the efficacy outcomes of this study should be interpreted cautiously.

Vortioxetine's multimodal action, influencing serotonergic, dopaminergic, and noradrenergic systems, may address the complex neurobiology of depression in PD more effectively than SSRIs, which may worsen motor symptoms or lead to apathy.

In fact, dysfunction of all aminergic systems has been shown in depressed PD patients, which in turn modifies the function of glutamate, GABA, acetylcholine, and opiate systems. This negatively impacts a variety of NMS, such as apathy, cognitive functions (prefrontal executive functions in particular), fatigue, and sleep disturbances. The role of vortioxetine in the treatment of depression in PD patients was recently confirmed in a Delphi survey conducted by Italian neurologists and geriatricians, who agreed that the tolerability and safety profile of antidepressant drugs should be considered as a significant criterion of selection. They also confirmed that vortioxetine may have some potential benefits on anhedonia and cognitive symptoms of depression in PD patients [22].

## CONCLUSION

Depressive symptoms are common in patients suffering from PD. Due to the pathophysiological mechanism, PD patients may experience worsening symptoms when treated with drugs that interfere with the catecholaminergic system. Some antidepressants can cause worsening tremors, while others can worsen all PD symptoms. This study was conducted to evaluate the tolerability of vortioxetine in PD patients, and the results show that the drug is well tolerated by these patients.

The open-label design of the study and the small sample size are limiting factors. However, as the study was designed to test tolerability, the sample size is sufficient, and the open-label design is acceptable. The efficacy results on depressive symptoms were secondary endpoints and should be taken with caution.

Vortioxetine seems to represent an innovative therapeutic option in the treatment of depression in PD because of its excellent safety and tolerability profile associated with a unique antidepressant mode of action. It most likely induces an increase of dopaminergic neurotransmission in brain structures usually associated with the development of depression. Future large-scale, double-blind, randomized trials are necessary to confirm these findings and fully characterize the clinical benefit of vortioxetine in this population.

## AUTHORS’ CONTRIBUTIONS

The authors confirm their contribution to the paper as follows: FS; LV: contributed to the research design and implementation, data analysis and manuscript writing. DB; FGR; CC: contributed to patient evaluations, performing tests for vital signs, data analysis manuscript review.

## Figures and Tables

**Fig. (2):**
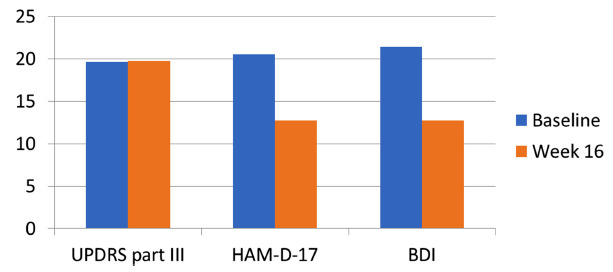
HAM-D17 total score, BDI total scale and UPDRS-III scale at the baseline and after 16 weeks of treatment (changes in HAMD-17 and BDI; *p*<0.001).

**Table 1:** Sociodemographic data, comorbidities, antiparkinsonian drugs at baseline (N = 20).

-	N = 20
Age	64.2 ± 10.3
Gender (N)	12 M, 8 F
Years of disease	6.25 ± 3.02
Years of depression	1.8 ± 1.05
Levodopa Equivalent Dose (LED)/DIE (mg/die)	656.75 ± 279.47
Treatment for PD (%):	-
Levodopa	100%
MAO-B inhibitor	90%
COMT inhibitor	55%
Dopamine agonist	50%
Amantadine	30%
H&Y	2.1 ± 0.52

**Table 2:** HAM-D17 total score, BDI total scale and UPDRS-III scale at the baseline and after 16 weeks of treatment.

**-**	**Mean**	**N**	**Standard Deviation**	**Standard Error of the Mean**
HAM-D-17 week 16	12.7500	16	4.71169	1.17792
HAM-D-17 baseline	20.5000	16	4.03320	1.00830
BDI week 16	12.7500	16	6.10464	1.52616
BDI baseline	21.4375	16	7.22928	1.80732
UPDRS III week 16	19.7500	16	4.00832	1.00208
UPDRS III baseline	19.6250	16	4.27200	1.06800

**Table 3:** Change in the HAM-D17 total score, BDI total scale and UPDRS-III scale from baseline to 16 weeks.

**-**	**Mean Change** **From Baseline**	**Standard ** **Deviation**	**Standard Error of the Mean**	**95% Confidence Interval of the Difference**		**Bilateral *P***
				**Lower**	**Upper**	
HAM-D-17 baseline HAM-D-17 week 16	-7.75000	4.04145	1.01036	-9.90354	-5.59646	**<.001**
BDI baseline BDI week 16	-8.68750	6.47785	1.61946	-12.13930	-5.23570	**<.001**
UPDRS -III week 16 UPDRS -III baseline	.12500	1.20416	.30104	-.51665	.76665	**.684**

## Data Availability

All the data and supporting information is provided within the article.

## LIST OF ABBREVIATIONS

BDI: Beck Depression Inventory
DDS: Dopa Dysregulation Syndrome
MAOIs: Monoamine Oxidase Inhibitors
NMS: Non-motor Symptom
PD: Parkinson’s Disease
SERT: Serotonin Transporter
SSRIs: Selective Serotonin Reuptake Inhibitors
TEAEs: Treatment-emergent Adverse Events

## STANDARDS OF REPORTING

CONSORT guidelines were followed.

## DECLARATION

This study was conducted independently by the center and received only partial unrestricted support from Lundbeck, Italy, which had no role in the design and conduct of the study, collection, management, analysis, and interpretation of data. The authors wrote the manuscript independently, and the preparation, review, approval, and decision to submit the manuscript for publication were made solely by the authors.
